# Role of double blind placebo controlled challenge test with wheat followed by exercise in patients suspected of wheat dependant exercise induced anaphylaxis (WDEIA)

**DOI:** 10.1186/2045-7022-1-S1-P73

**Published:** 2011-08-12

**Authors:** Mauritus Van Maaren, Nicolette De Jong, Frans Mertens, Henk Stam, Roy Gerth van Wijk

**Affiliations:** 1 Erasmus University Medical Center, Allergology, Rotterdam, Netherlands; 2 Erasmus University Medical Center, Respiratory Physiology, Rotterdam, Netherlands

## Background

Double blind placebo controlled food challenge tests (DBPFC) are essential for diagnosing food allergy. However, open food challenges followed by exercise are used to establish the diagnosis of Wheat Dependent Exercise Induced Anaphylaxis (WDEIA). The presence of specific IgE to ω-5 gliadine ≥ 0.89 Ku/L was highly predictive for WDEIA in one study.

## Methods

Patients characterized by a history of at least two anaphylactic reactions during exercise within four hours following ingestion of wheat and the presence of specific IgE to ω-5- gliadin ≥.0.89 Ku/L were asked to undergo a DBPFC with wheat masked in a pancake followed by an exercise test . Patients with negative responses were asked to participate in an open challenge test thereby consuming a 2.5 fold higher amount of wheat compared to the dose ingested in the blinded test.

## Results

Eight patients aged 34 – 57 years, fulfilling the inclusion criteria were willing to participate in a double blinded challenge – exercise test. One patient had a history of consistent reactions during exercise after wheat ingestion; in others reactions varied. Most patients recognized concomitant factors such as intake of alcohol or sudden temperature change during exercise induced reactions. None reacted in the double blinded test. Five were willing to participate in an open challenge test. Two reacted in this open challenge test. They revealed higher levels of specific IgE to ω-5 gliadin (37.5 and 14.0 Ku/l respectively) compared to the other 3 patients (6.6, 3.97 and 2.17 Ku/L respectively)

## Conclusions

A DBPCFC is not suitable to establish the diagnosis WDEIA probably because of the low maximum dose of wheat that can be masked in a food matrix and the presence of concomitant factors in real life. The presence of specific IgE to ω-5 gliadine 0.89 kU/L is not sufficient to establish the diagnosis of WDEIA.

